# Mixed Reality as a Teaching Tool for Medical Students in Neurosurgery

**DOI:** 10.3390/medicina59101720

**Published:** 2023-09-26

**Authors:** Arturo Silvero Isidre, Hendrik Friederichs, Michael Müther, Marco Gallus, Walter Stummer, Markus Holling

**Affiliations:** 1 Department for Neurosurgery, University Hospital Münster, 48149 Münster, Germany; 2 Medical School OWL, Bielefeld University, 33615 Bielefeld, Germany

**Keywords:** medical students, medical education, mixed reality, computer simulation, neurosurgery

## Abstract

*Background and Objectives*: Simulation-based learning within neurosurgery provides valuable and realistic educational experiences in a safe environment, enhancing the current teaching model. Mixed reality (MR) simulation can deliver a highly immersive experience through head-mounted displays and has become one of the most promising teaching tools in medical education. We aimed to identify whether an MR neurosurgical simulation module within the setting of an undergraduate neurosurgical hands-on course could improve the satisfaction of medical students. *Materials and Methods*: The quasi-experimental study with 223 medical students [120 in the conventional group (CG) and 103 in the MR-group (MRG)] was conducted at the University Hospital Münster, Münster, Germany. An MR simulation module was presented to the intervention group during an undergraduate neurosurgical hands-on course. Images of a skull fracture were reconstructed into 3D formats compatible with the MR-Viewer (Brainlab, Munich, Germany). Participants could interact virtually with the model and plan a surgical strategy using Magic Leap goggles. The experience was assessed by rating the course on a visual analog scale ranging from 1 (very poor) to 100 (very good) and an additional Likert-scale questionnaire. *Results*: The satisfaction score for CG and MRG were 89.3 ± 13.3 and 94.2 ± 7.5, respectively. The Wilcoxon rank-sum test showed that MR users (Mdn = 97.0, IQR = 4, *n* = 103) were significantly more satisfied than CG users (Mdn = 93.0, IQR = 10, *n* = 120; ln(W) = 8.99, *p* < 0.001) with moderate effect size (${\hat{r}}_{biserial}$ = 0.30, CI95 [0.15, 0.43]), thus indicating that the utilization of MR-simulation is associated with greater satisfaction. *Conclusions*: This study reports a positive response from medical students towards MR as an educational tool. Feedback from the medical students encourages the adoption of disruptive technologies into medical school curricula.

## 1. Introduction

Many students start medical school with a high interest in neurosciences [1]. However, the fraction of medical students who actually consider neurosurgery a future career varies among countries and institutions [2,3]. This could be due to negative perceptions of neurosurgery that intimidate students from potentially pursuing it as a career [3].

The increasing emphasis on general practice principles in the undergraduate curriculum has led to less exposure towards surgical subspecialties such as neurosurgery [4]. Therefore, a high-quality educational experience is needed to promote undergraduate neurosurgical education [5].

This need has increased due to the coronavirus disease 19 (COVID-19) pandemic [6]. Since then, strict social distancing rules have been enforced worldwide, negatively affecting medical education [7]. Therefore, to maintain the quality of medical education in the circumstances of the COVID-19 pandemic, medical educators promoted pedagogical innovations [8].

One of medical education’s most promising teaching tools is extended reality (XR) technology. This term includes all immersive technologies, such as virtual reality (VR), augmented reality (AR), and mixed reality (MR) [9]. Although the terms “mixed reality” and “augmented reality” are sometimes used interchangeably, MR can be conceptually seen as an extension of AR technology [10] and delivers a highly immersive experience through head-mounted displays (HMDs). In addition, it provides an interactive and dynamic form of simulation in which objects of a physical and virtual environment are combined [11]. It has been used in neurosurgery to develop technical competencies, such as surgical skills, or to conceptualize complex 3D anatomic relationships [12]. Another essential aspect that favors the use of this technology is that learners find this technology engaging and entertaining for learning purposes [13,14].

Simulation-based medical education (SBME) is an ever-evolving part of medical education that has demonstrated remarkable efficacy in teaching basic surgical skills and general medical scenarios, outshining traditional teaching methodologies in numerous ways. A recent systematic review has reaffirmed that, when used in conjunction with current curricula, SBME not only enhances students’ performance-based knowledge transfer in the short term, but also improves knowledge retention over the long term. All studies included in the review reported positive effects on knowledge acquisition, with three of them demonstrating improved retention [15].

Simulation is an incredibly beneficial tool for undergraduate medical education. It provides a controlled learning environment that allows students to repeat relevant content and structured exercises, leading to successful learning outcomes [16]. This type of environment is ideal for providing immediate feedback, which is a crucial aspect of medical education [17]. Feedback and practical involvement are also highlighted in qualitative studies as important factors in the clinical scenario [18]. Simulated training offers students the chance to practice patient care outside of the hospital setting and improve their abilities in accordance with adult learning and reflective practice principles [17]. Research shows that repetition opportunities and immediate feedback are both essential components of simulation-based medical education programs [19], making simulators a valuable addition to practical medical education [20]. Simulation-based learning strategies are also effective in surgical education, with a systematic review suggesting that they can improve students’ skills and knowledge while promoting the safety and efficiency of future surgical professionals [21].

Furthermore, undergraduate SBL provides valuable and realistic educational experiences in a safe environment, enhancing the current teaching model [22,23]. Such simulators can be classified into physical models, virtual reality, and mixed reality [24].

Therefore, we aimed to identify whether an MR neurosurgical simulation module within the setting of an undergraduate neurosurgical hands-on course could improve the satisfaction of medical students with no previous practical neurosurgical experiences.

## 2. Materials and Methods

### 2.1. Setting and Subjects

In Germany, the medical program typically lasts for 6.25 years and is split into two sections: preclinical (the first two years) and clinical (the last four years). During the practical year, which is the final year, students rotate through various hospital departments to gain hands-on experience. The National Competency-Based Learning Objectives Catalogue for Medicine (NKLM) was introduced in 2015 to outline the competencies required for medical students [25]. The NKLM now includes the acquisition of fundamental applied abilities. However, most of the curriculum still focuses on knowledge-based material, similar to the international approach [26]. In our particular setting, neurosurgical teaching aims to provide students with a basic knowledge of applied neuroanatomy, identification of clinical symptoms and syndromes, and practical insight into basic neurosurgical skills.

The practical aspects of training can be customized by the implementing departments. The teaching materials are distributed for this purpose through mandatory evaluations. Therefore, there is a competitive interest in high-quality training in terms of content, practicality, and demand.

Over the past decade, we have introduced several teaching formats to achieve our educational objectives. These include independent lecture series, which run concurrently and cover all crucial facets of neurosurgery by experts in the field. The content and personnel involved in the lectures have remained consistent throughout the period under review. Due to the COVID-19 pandemic, the lectures were delivered in a live video format.

### 2.2. Study Design

The quasi-experimental study was conducted at the University Hospital of Münster (Münster, Germany) from January 2019 to June 2022. All medical students were invited to participate in the hands-on course in the 8th semester. A total number of 260 students were enrolled during this period. In total, 223 medical students who agreed to participate completed the questionnaire and were included in the study, yielding a response rate of 85.77%.

The students were non-randomly assigned into two groups: the conventional group from January 2019 to June 2020 (CG, *n* = 120) and the MR group from July 2020 to June 2022 (MRG, *n* = 103). Both groups underwent their training in the same educational environment during the same semester during the COVID-19 pandemic. Training was performed in similar group sizes, in the same room, at the same daytime and students received teaching by the same supervision/trainee ratio. Furthermore, students were assigned to the cohorts independently of their previous grades.

### 2.3. Hands-On Course-Simulation Settings

**Figure A1:**
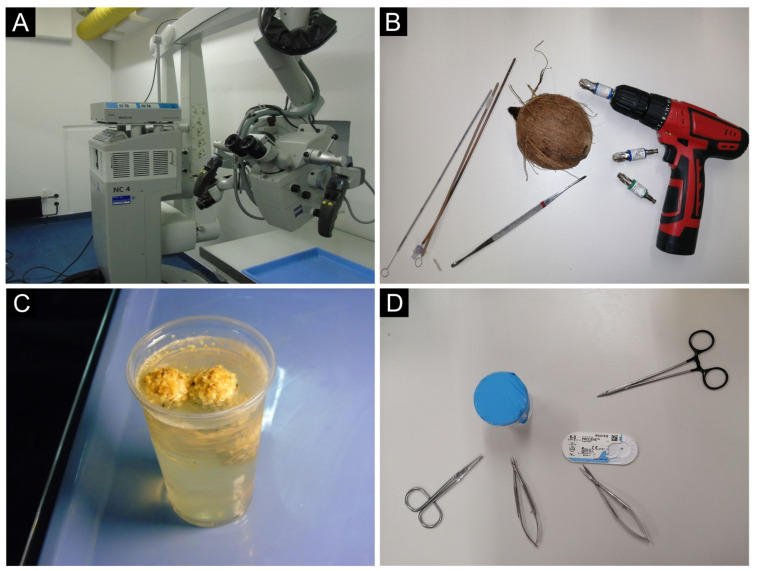
Neurosurgical hands-on course. (**A**) Training station at the University Hospital Münster with four OR microscopes. (**B**) Coconut to practice trepanation. (**C**) Bonbons in jelly to practice microsurgical resection of metastases. (**D**) Suturing latex gloves to practice dural-repair.

The one-day medical student hands-on course is conducted annually at the University Hospital Münster (Münster, Germany). The hands-on course is integrated in the curricular activities. It is divided into two parts. First, the medical students are trained in the basics of systematic neurosurgical/neurological examinations and learn the most common neurological diseases at the patient’s bedside. In the second part, exercises with neurosurgical instruments (Aesculap AG, Tuttlingen, Germany), an endoscope (Karl Storz GmbH & Co, Veitshöchheim, Germany) and microscopes (Carl Zeiss AG, Oberkochen, Germany) are carried out on various simulation-models in our skills lab [27,28] (Figure A1). To ensure an optimal supervision trainee ratio, this was at least 1:4.

Unlike the CG, participants in the MRG could interact virtually with the MR simulation model and plan a surgical strategy.

### 2.4. Prepared Case

**Figure 1:**
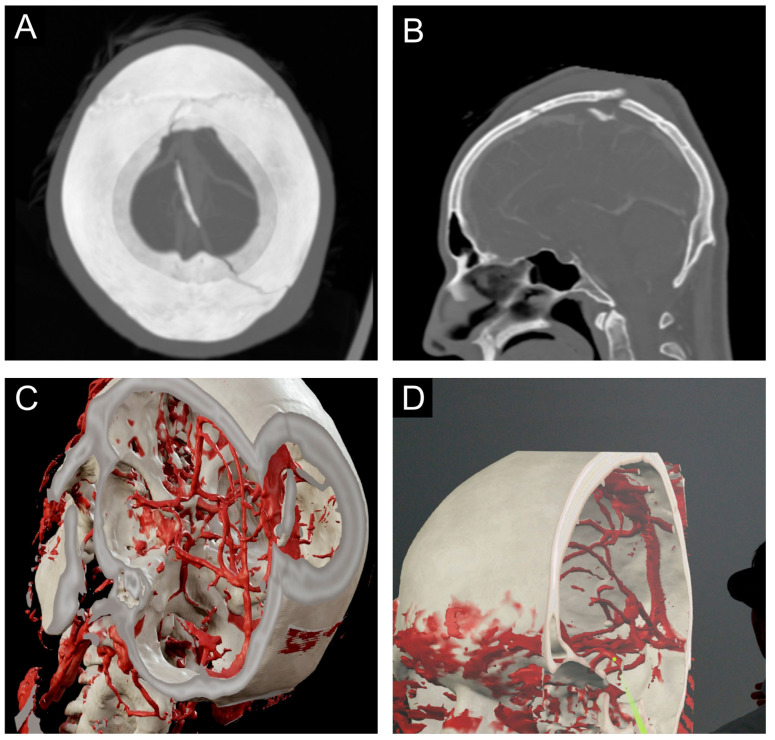
MR-simulated case: (**A**) axial-CT Image, (**B**) sagittal-CT image, (**C**) bone and vessel segmentation of a CT-angiography, and (**D**) screenshot visualizing the participants’ view.

The MR-simulated case was a 29 years old patient attacked with an axe, resulting in traumatic brain injury with a complex skull fracture. The diagnostic imaging shows a displaced osseous fragment in the superior sagittal sinus, with possible injury and stenosis (Figure 1A,B).

### 2.5. Technical Setup

This study’s virtual 3D simulation model was reconstructed from the computed tomography (CT) angiography scan. The CT imaging was performed on a Siemens SOMATOM Force (München, Germany) at the Department of Radiology at the University Hospital of Münster. The standard trauma protocol at our department includes a 1 mm slice thickness CT-skull and CT-angiography of the cerebral arteries. All in-house imaging was stored on the system server in Digital Imaging Communication in Medicine (DICOM) format.

The DICOM volumetric data were segmented with Brainlab Digital O.R.-Platform (Brainlab, Munich, Germany) (Figure 1C). The regions of interest (ROI) were chosen according to their importance for preoperative planning.

### 2.6. MR Interaction

Pre-briefing and debriefing sessions were held for the MRG (Figure 1D). The participants had the opportunity to interact with the 3D model for 5–10 min using immersive headsets (Magic Leap Inc., Plantation, FL, USA) (Video S1). They were equipped with a controller which allowed rotation of the model. In addition, the patient’s 2D MRI scan could be visualized in the background.

### 2.7. Data Collection

To evaluate the level of satisfaction, participants assessed their experience by rating the course on a visual analog scale ranging from 1 (very poor) to 100 (very good). The MRG was also asked about this technology’s usefulness and digital implementation. They had to fulfill another optional questionnaire based on a Likert scale with answers from 0 (minimum) to 10 (maximum) concerning specific aspects and anchors:

- Please give a general assessment of the benefit of the augmented/virtual reality technology used—scale: 1 (very high)–10 (very low)
- Should more or less time be invested in the use of augmented/virtual reality technology in the future?—Scale: 1 (more)–10 (less)
- Do you consider the augmented/virtual reality technology used to be more important than the opportunity to learn about practical microsurgical aspects?—Scale: 1 (more important)–10 (less important).

### 2.8. Statistical Analysis

The study used R [29] in RStudio IDE (Posit Software 2023.03.0, Boston, MA) with the tidyverse package [30] to conduct statistical analysis and to create figures. The mean, standard deviation (SD), median (Mdn), and interquartile range (IQR) of the Likert-type questions were calculated. The Wilcoxon rank-sum test with continuity correction as a non-parametric test on two independent samples was used to compare the groups, with effect sizes analyzed using rank-biserial correlation. An r of ≥0.1 was considered a small effect size, ≥0.3 medium, and ≥0.5 large. *p*-value ≤ 0.05 was considered statistically significant [31]

## 3. Results

The mean age of the participants was 25.33 years [age range 22–39 years]. Of the participants, 149 were females (66.96%).

**Figure 2:**
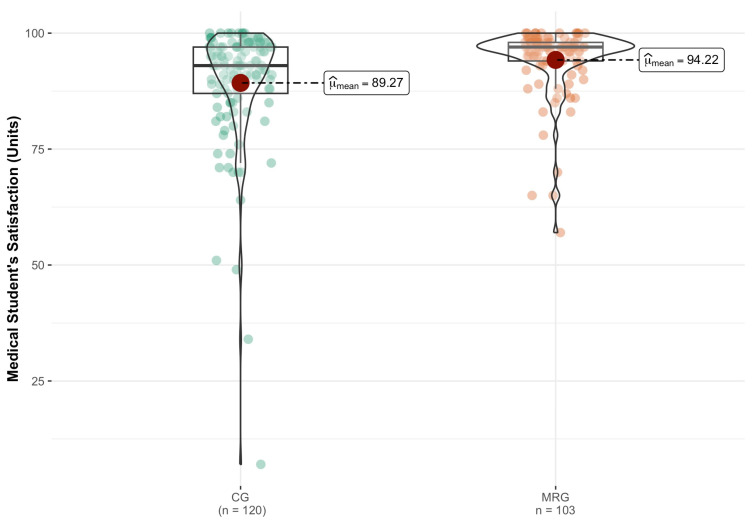
MR influence on the medical student’s satisfaction. Scale used: the highest score is 100, and the lowest is 1. *p* < 0.001.

Figure 2 shows the satisfaction score of the groups. The mean and SD for the CG and MRG were 89.3 ± 13.3 and 94.2 ± 7.5, respectively (Figure 2). The Wilcoxon rank-sum test showed that MR users (Mdn = 97.0, IQR = 4, *n* = 103) were significantly more satisfied than CG users (Mdn = 93.0, IQR = 10, *n* = 120; ln(W) = 8.99, *p* < 0.001) with moderate effect size (${\hat{r}}_{biserial}$ = 0.30, CI95 [0.15, 0.43]), thus indicating that the utilization of MR-simulation is associated with greater satisfaction.

MRG participants were asked to answer additional questions, which was not mandatory. The lowest response rate was 87.4% (Table 1).

## 4. Discussion

This research study entailed an assessment of medical students’ contentment subsequent to the integration of a mixed reality (MR) simulation module within our neurosurgical hands-on course. Comparing the satisfaction levels of participants in the MR group (MRG) with those in the conventional group, notable distinctions emerged, illuminating the discernible impact of MR simulation within a pedagogical context. Additionally, the feedback solicited from students in the MRG was overwhelmingly affirmative. The majority of participants experienced MR as enjoyable, beneficial, and novel, thus advocating for the further investment of time in MR-based learning activities. This is consistent with several studies documenting the positive impact of immersive technology with overall positivity and higher satisfaction in learning, self-efficacy, and engagement [32]. These positive reports have also led to interest by medical educators as well as students to incorporate MR applications into medical school curricula [33,34].

Although XR technologies, such as MR, have been considered as promising novel tools to enhance the quality of medical student teaching, data evaluating their feedback is missing [5,9,35,36]. Although the individual merits of MR technology are recognized, it is essential to underscore that a conclusive consensus remains elusive in the academic literature concerning its impact on improving students’ recall and retention abilities [37]. However, it is noteworthy that meta-analyses and systematic reviews predominantly indicate positive outcomes, showcasing its potential to enhance students’ overall learning experience, as well as skill and knowledge acquisition [13,14,38].

The COVID-19 pandemic has severely impacted all aspects of medical education. However, surgical trainees face even more significant challenges [8,39]. Neurosurgical trainees reported decreased operative volumes, reduced procedure participation, and less time in the OR [40]. To bridge this education gap, the adoption of digital technologies in medical training has been accelerated [8,41]. The COVID-19 restrictions forced educators to rethink solutions to maintain the quality of medical training.

Barteit et al. [11] found that MR-based HMDs may significantly improve learning outcomes. In addition, 3D models, with HMDs providing 360° views, enable the understanding of complex organ structures. Therefore, it is a revolutionary tool in preoperative neurosurgical planning, allowing for patient-specific approaches and training programs related to cranial surgery [42,43]. However, consistent with our results, the current literature suggests that MR technology cannot replace cadavers or physical simulation models in neurosurgery [42]. Chawla et al. [22] revealed that haptic sensation was a fundamental limitation to the success of VR models in neurosurgery. Moreover, AR and MR may become effective tools in conjunction with cadaveric or physical simulation models to improve efficiency and long-term retention [10,44].

Even though neurosurgery across the world remains a competitive field, there are countries where medical students’ interest in neurosurgery has decreased markedly; therefore, help is needed recruiting trainees [3,23]. Medical students choose their careers according to the specialties they have been exposed to [3]. Yang et al. [45] found that individual factors, including academic interest and competencies, considerably impact students’ subspecialty choice, with the extent of their influence values of 75.29% and 55.15%, respectively. Previous studies indicated that early specialty exposure in medical education might arouse these factors; for that reason, early neurosurgical exposure and maintaining exposure are indispensable to sustaining interest [1,2,3].

High-quality neurosurgical SBL hands-on courses for medical students are a way of countering such challenges [23]. This increases the understanding of neurosurgery and motivates students to expose themselves to clinical neurosurgery [46].

### Limitations

Although the researcher reiterated to students to remain honest, the Hawthorne effect may introduce bias by the participants knowing their attitudes were being assessed [37]. Also, it is difficult to conclude the data of the MRG because of the small sample size returning questionnaires, and students that were interested in MR were more willing to complete the questionnaire. Another disadvantage of quasi-experimental studies is that the participants are not randomly assigned, which makes them more likely to have systematic bias.

## 5. Conclusions

This study reports a positive response from medical students toward introducing MR as an educational tool. The MR may provide an effective complementary tool to expose medical students to neurosurgery. The feedback from medical students encourages the adoption of disruptive technology into medical school curricula.

## Figures and Tables

**Table 1:** Questionnaire of the MRG.

Questions	Participants (%)	Mean ^1^ ± SD
Please give a general assessment of the benefit of the mixed reality technology used	90 (87.4%)	8.96 ± 1.55
Should more time be invested in using augmented/virtual reality technology in the future?	91 (88.3%)	8.56 ± 1.77
Do you think the mixed reality technology used is more important than the opportunity to get to know practical microsurgical aspects?	90 (87.4%)	6.57 ± 2.72

^1^ Likert-scale used: the highest score is 10, and the lowest is 1.

## Data Availability

The datasets used and/or analyzed during the current study are available from the corresponding author upon reasonable request.

## Supplementary Materials

The following supporting information can be downloaded at: https://www.mdpi.com/article/10.3390/medicina59101720/s1, Video S1: Interaction with the segmented 3D-Model (Brainlab, Munich, Germany) using immersive AR-headsets (Magic Leap Inc., Plantation, FL, USA).
